# Substance P scavenger enhances antioxidant defenses and prevents prothrombotic effects on the rat lung after acute exposure to oil smoke

**DOI:** 10.1186/1423-0127-16-58

**Published:** 2009-07-06

**Authors:** Li Ping-Chia, Lai I-Ju, Lin Yu-Ching, Chang Li-Ching, Chen Wen-Chung

**Affiliations:** 1 Department of Occupational Therapy, I-Shou University, No. 8 E-Da Road, Jiau-Shu Tsuen, Yan-Chau Shiang, Kaohsiung County 824, Taiwan, R.O.C; 2 Department of Medical Nutrition, I-Shou University, No. 8 E-Da Road, Jiau-Shu Tsuen, Yan-Chau Shiang, Kaohsiung County 824, Taiwan, R.O.C; 3 Department of Physical Medicine and Rehabilitation, E-DA Hospital, No. 1 E-Da Road, Jiau-Shu Tsuen, Yan-Chau Shiang, Kaohsiung County 824, Taiwan, R.O.C; 4 Department of Pathology, National Cheng Kung Univeristy Hospital, No. 138 ShengLi Rd., Tainan City, Taiwan R.O.C

## Abstract

**Background:**

Airborne particulate matter, from cooking oil, smoking, engine exhaust and other sources, is associated with the development of atherosclerosis and myocardial infarction. In order to explore the cellular and molecular events following exposure of rats to lard oil smoke, we measured the generation of reactive oxygen species (ROS), substance P, cellular adhesion molecules, and thrombosis in relation to inhibitors of substance P, the NK-1 receptor, and antioxidants.

**Methods:**

Rats were exposed to oil smoke for 120 min with or without 20 min pretreatment with lovastatin (substance P scavenger), L733060 (NK-1 receptor antagonist), vitamin E (antioxidant) or catechins (antioxidant). The levels of substance P and ROS were measured. Histological studies observed ROS damage in the form of HEL adducts. The prothrombotic effects of oil smoke exposure were measured by experimental induction of thrombosis in vivo.

**Results:**

Oil smoke exposure significantly increased substance P levels, ROS levels, ROS damage (HEL adduct levels), and the size of experimentally induced thrombi. The pretreatments reduced all of these effects of oil smoke exposure; at many time points the reductions were statistically significant.

**Conclusion:**

We established a connection between oil smoke exposure and thrombosis which involves substance P and its receptor, the NK-1 receptor, and ROS. This study helps establish a mechanistic explanation of how airborne particulate matter can increase the risk of cardiovascular illness.

## Background

A variety of chemical reactions occur while high temperature (150–400°C) cooking oils (e.g. sunflower oil, soybean oil, or lard) are being heated before food is added to the pan [1-3]. Several volatile chemicals such as formaldehyde, benzene, acetaldehyde and acrolein are present in fumes generated from frying and are thought to be associated with contents of cooking oil and foods [1-3]. Particulate air pollution is also generated and is associated with cardiovascular disease and myocardial infarction [4]. Indoor inhalation of atmospheric particulate matter (APM) from cooking has been associated with respiratory and cardiovascular diseases [5,6]. APM is as an important risk, particularly for cooks, in areas where pan-fry cooking is popular.

Inhalation of the APM from cooking has been suggested to impact on the non-adrenergic and non-cholinergic (NANC) nervous system, leading to changes in breathing pattern, smooth muscle, or blood vessels in order to regulate visceral motility and blood flow [7]. APM from cooking may affect the cardiovascular system through inflammatory mediators produced in the lungs and released into the circulation [7]. NANC stimulation and overflow of inflammatory mediators from the lungs into blood may constitute an alternative and a complementary explanation for the acute cardiovascular events after exposure to APM [7].

Substance P (SP) is an NANC signaling molecule that induces a range of neuroimmunogenic effects on target smooth muscle or parasympathetic ganglia cells expressing neurokinin-1 or neurokinin-2 receptors (NK-1R, NK-2R) [8-12]. Neurogenic airway inflammation comprises bronchoconstriction, microvascular plasma leakage, neutrophil recruitment and inflammatory mediator synthesis. SP-mediates neutrophil adhesion to bronchial epithelial and endothelial cells and further augments the proinflammatory response and oxidative stress via production of intercellular adhesion molecule-1 (ICAM-1), chemokines/cytokines, arachidonic acid products, and reactive oxygenated species (ROS)/nitric oxide (NO) derivatives such as hydroxyl radicals and superoxides [10,12,13]. Recently, we have shown that oil smoke exposure (OSE) causes a time-related release of SP in bronchoalveolar lavage fluid (BALF) 0–8 h after OSE in rats [7]. Substantial elevations in tumour necrosis factor-α (TNF-α) and IL-1 beta gene expression, superoxide anion levels, epithelial cell necrosis and alveolar hypoventilation after OSE were also noted [7].

SP and NK1R have also recently been recognized to be involved in thrombosis [14-18]. SP can increase the chance of thrombosis. And, thrombosis can lead to myocardial infarctions and strokes [15]. Thus, SP signaling may explain APM exposure can lead to increased incidences of thrombosis and myocardial infarctions [4].

The precise role that SP plays in mediating neurogenic inflammation through interaction with NK-1R and consequent ROS and ICAM-1 formation following OSE was clarified in our previous study [7]. The goal of this study was (1) to evaluate the oxidative stress enhanced thrombus and its relationship to the pulmonary inflammation accompanying exposure following OSE and (2) to investigate the mechanism of this phenomenon.

## Methods

### Animals

Male Sprague-Dawley rats (12 to 14 weeks-old, weighing 200 to 250 g) were housed at the Experimental Animal Center, I-Shou University, in a temperature controlled environment with light from 0700 to 1800 h. Animal care and experimental protocols were in accordance with guidelines of the National Science Council of the Republic of China (NSC1997). All experiments were approved by the Institutional Animal Care and Use Committee (IACUC) of I-Shou University, Kaohsiung, Taiwan (IACUC Protocol no. AUP-96-55-001 and Approval no. IACUC-ISU-96014).

Rats were anesthetized with 1.2 g/kg subcutaneous urethane (Sigma, St. Louis, MO, USA). Then, the trachea was cannulated caudal to the larynx (PE-200), and femoral vein catheters (PE-50, Clay-Adams, Parsippany, NJ, USA) were inserted to facilitate blood sampling and drug administration. Blood vessel patency was maintained by intravenous saline infusion at 1.2 ml/min via an infusion pump (CH-4103; Infors, Bottmingen, Switzerland). Femoral arterial catheters were placed for measurement of arterial blood pressure (ABP). This was recorded using a polygraph (ML845 PowerLab 4/25 T system, ADInstruments, Sydney, Australia).

### Pretreatment with substance P scavenger, neurokinin-1 receptor antagonist, or antioxidants

On the experimental day, groups of rats were pretreated with either: L733060, an NK-1 receptor antagonist (Sigma); lovastatin, a cholesterol or substance P scavenger (Sigma); vitamin E, an H_2_O_2 _scavenger (Sigma); or catechins, which are O_2 _^- ^and H_2_O_2 _scavengers. We administered: 1 μg/kg L733060 dissolved in 10% dimethyl sulphoxide (DMSO) in 0.9% saline; 4 mg/kg lovastatin dissolved in 50% DMSO in PBS; 2.5 mg/kg vitamin E (1 mg/mL in 0.9% saline); or 2.5 mg/kg catechins (consisting of 328 mg/g epigallocatechin gallate, 132 mg/g epicatechin, 108 mg/g epigallocatechin, 104 mg/g galloctechin, and 44 mg/g catechin; Numen Biotech, Taipei, Taiwan) in 0.9% saline. All drugs were administered as a bolus via the femoral catheter. Atropine (1 mg/mL/kg) was given 20 min before OSE to inhibit cholinergic innervation of the airway nervous system.

### Compulsory oil smoke exposure (OSE)

Compulsory oil smoke exposure followed our previously published procedure [7] with modifications. Briefly, in our custom-built apparatus, oil was heated to produce smoke, the smoke was passed into an artificial respirator machine, and cooled smoke was forced through tracheotomies into the lungs of anesthetized rats. To produce the smoke, 500 mL of lard was placed in a ceramic crucible and burned in the stainless steel smoke chamber. This thermal furnace system produced a consistent high temperature of 350 ± 6°C. The effluent oil smoke generated was vacuum transferred through an inverted funnel canopy into the sealed chamber of the artificial respirator. The smoke was cooled in the artificial respirator device, and the mean smoke temperature for all exposures was 32.6 ± 1.7°C. The artificial respirator was attached to the trachea of urethane-anesthetized, cannulated rats, and then cooled oil smoke was artificially respirated through the rats at a continuous flow (dynamic) rate of 2 L/min for 120 min (compulsory oil smoke exposure; OSE). Control rats were artificially respirated with ambient room air only.

Rats that survived the OSE procedure were sacrificed immediately and up to 24 h following OSE to assess lung injury (specific details of this follow). A further subset of (non-pretreated) rats was exposed to OSE for 0 and 120 min and killed thereafter between 0 and 24 h for assessment of SP levels in plasma and ROS levels in blood.

### Measurement of substance P

SP levels in plasma supernatant were quantified using a commercial enzyme immunoassay kit (Cayman Chemical, Ann Arbor, MI, USA) following the manufacturer's instructions. The concentration of SP in plasma was expressed in pg/mL.

### Measurement of reactive oxygen species

The effect of treatments on plasma ROS levels was measured using the lucigenin-enhanced chemiluminescence method. Lucigenin reacts almost exclusively with ROS under optimal laboratory conditions and is commonly used to increase the sensitivity of cellular chemiluminescence assays [19]. For this measurement, 0.1 mL of PBS (pH 7.4) was added to each 0.2 mL arterial blood sample. The chemiluminescence of the sample was then measured in the dark chamber of the chemiluminescence analyzing system. After a 100-s background level luminescence determination, 0.5 mL of 0.1 mM lucigenin solution in PBS (pH 7.4) was added to the sample and chemiluminescence was monitored continuously for an additional 600 s. The total amount of chemiluminescence was calculated by integrating of the area under the 600 s chemiluminescence curve and subtracting the background luminescence level. The assay was performed in duplicate for each sample, and the results expressed as chemiluminescence counts/10 s.

### Measurement of damage caused by reactive oxygen species

Hexanoyl-lysine (HEL), a biomarker for ROS damage, is generated when ROS oxidize lipids and those lipid hydroperoxides modify proteins at lysine residues; HEL is detectable by an anti-HEL antibody [20].

To determine if HEL had been generated at various times following OSE stimulation, lungs were removed and fixed by perfusing 10% buffered formalin through the trachea at a pressure of 30 cm H_2_O for 24 h. The lungs were then dehydrated through a series of ethanol solutions, cleared with methylsalicylate, and embedded in paraffin. Sections of the lung tissue (5 μm) were mounted on glass slides and stained with hematoxylin and eosin for examination by light microscopy. For immunohistochemical analysis, the sections were first treated with blocking buffer for 15 min to block nonspecific background binding. Primary rabbit-antiserum (anti-VCAM-1 or anti-HEL, 1:1000) was then applied overnight at 4°C. The anti-VCAM-1 was obtained from R&D system and the monoclonal anti-HEL antibody (Kato, et al., 2000) was from JaICA (Fukuroi City, Japan). After rinses in PBS, sections were treated with the Zymed Histostain kit (Zymed Laboratories, South San Francisco, CA, USA). This included secondary biotinylated goat anti-rabbit serum followed by horseradish peroxidase-conjugated streptavidin and subsequent development in a diaminobenzidine-substrate solution, yielding a permanent reddish brown reaction product. All slides were permanently coverslipped with crystal-mount.

The presence of HEL was also determined by immunostaining western blots of proteins from the right lung following OSE. For protein analysis, lung samples were homogenized with a pre-chilled mortar and pestle in extraction buffer (10 mM Tris-HCl [pH 7.6], 140 mM NaCl, 1 mM phenylmethylsulfonyl fluoride, 1% NP-40, 0.5% deoxycholate, 2% β-mercaptoethanol, 10 μg/mL pepstatin A, and 10 μg/mL aprotinin). The homogenate was maintained at 4°C for 30 min, and then centrifuged at 12,000 *g *for 12 min at 4°C to collect the supernatant. Protein concentrations were determined by BioRad protein assay (BioRad Laboratories, Hercules, CA, USA). The protein samples (30 μg total protein per lane) were separated on 12.5% SDS-polyacrylamide gel electrophoresis (PAGE) separation gels in the absence of urea, stained with Coomassie brilliant blue, and transferred to nitrocellulose filters. Immunoreactive bands were detected by incubating with primary antibodies for HEL for approximately 4 h, followed by an alkaline phosphatase-conjugated secondary antibody for 1 h, and finally developed with nitroblue tetrazolium and a 5-bromo-4-chloro-3-indolyl phosphate toluidine salt (Roche Diagnostic, Mannheim, Germany) stock solution for 30 min at room temperature.

### Measurement of thrombosis

To measure the effects of OSE on thrombosis formation we followed the method of Nemmar et al. [18]. Briefly, an anesthetized rat was intravenously infused with a solution of Rose Bengal and a section of the femoral vein was surgically exposed. This vein was then exposed to green light (540 nm) for 2 min, which causes the Rose Bengal to produce ROS that cause an endothelial lesion and then thrombosis.

The kinetics of thrombus generation were monitored using a microscope video camera. MetaMorph imaging software was used for image processing on a preset scale of arbitrary units ranging from 0 to 4,095. All images were processed to the same scale, and the average light intensity of each field was obtained. The size of the thrombus was expressed in arbitrary units (A.U.) as the total area under the curve of light intensity plotted against time.

### Statistical methods

Normally distributed continuous variables were compared by one-way analysis of variance (ANOVA). When a significant difference between groups was apparent, multiple comparisons of means were performed using Scheffe's test. Data are presented as means ± SE. All statistical assessments were two-sided and evaluated at the 0.05 level of significant difference. Statistical analyses were performed using SPSS 15.0 statistics software (SPSS Inc, Chicago, IL).

## Results

### Effects of oil smoke exposure

**Figure 1:**
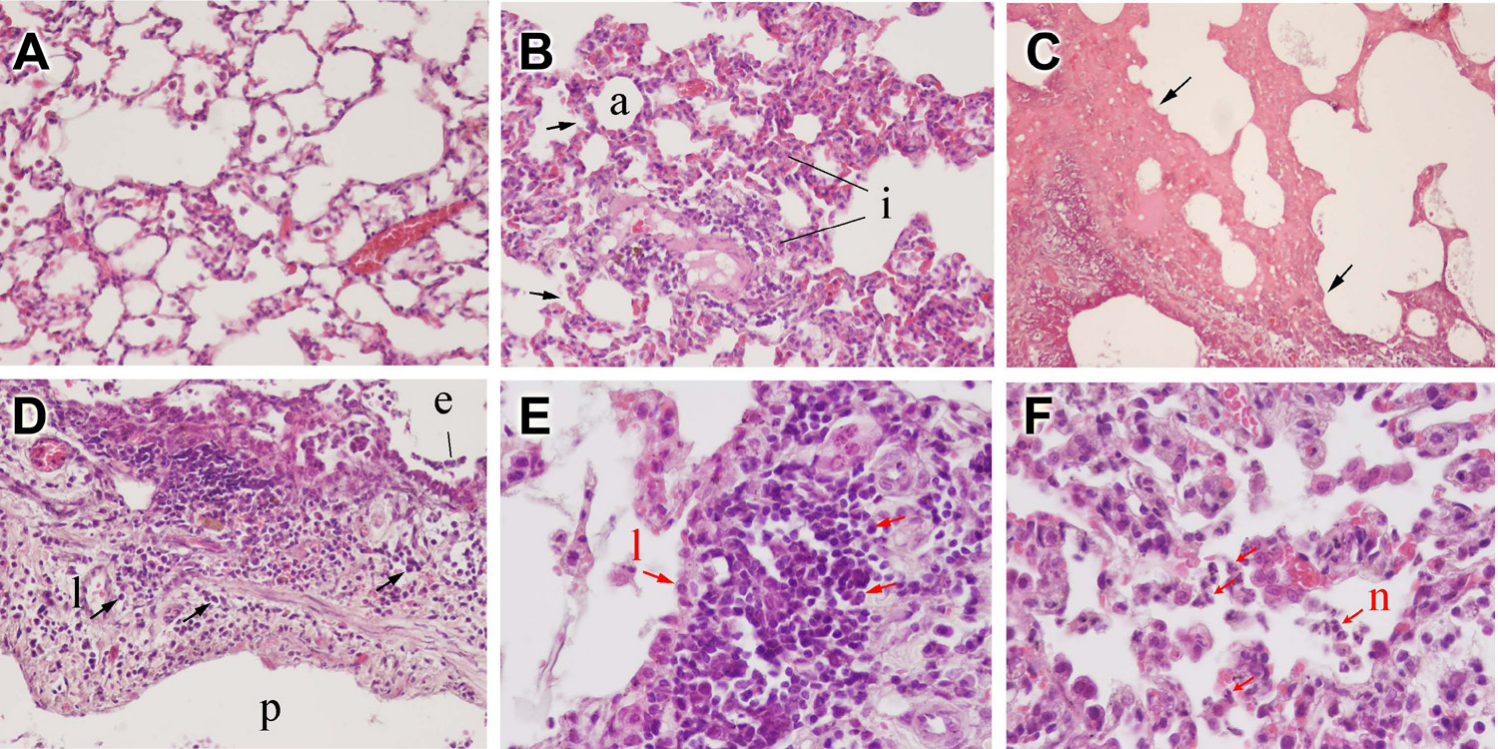
**Representative histopathological slides from lung tissue following oil smoke exposure (OSE)**. (A) The trachea shows normal structure without inflammation. (B) Peripheral lung tissue shows interstitial pneumonitis with infiltration of lymphplasmacytic cells after oil smoke exposure. (C) Peribronchiolar hemorrhage and sloughing of bronchiolar epithelium. (D) Heavy infiltration of lymphocytes and some histiocytes and neutrophils in peribronchiolar areas. (E) The lung tissue shows mild lymphoplasmacytic infiltration in the submucosal area. (F) Neutrophilic infiltration in the alveolar space and interstitium.

Compared to control rats exposed to ambient air, the rats given OSE showed an increasing plasma SP level until 8 h following compulsory OSE, then gradually reducing (Table 1). There was also a concomitant increase in ROS in the plasma from the OSE group between 0 and 8 h after OSE, then reducing to a plateau (Table 1). There were significant differences in substance P and plasma ROS between OSE and control groups at all time points following OSE (*P *< 0.05). Histologically, oil smoke exposure caused microscopic hemorrhaging and the sloughing of cells around the bronchi of rat lung (Fig. 1C); at the same time, the amount of inflammatory cells observed in the lung tissues (Fig. 1B, D, E, F) were increased compared to mnoraml rat lungs (Fig. 1A).

**Table 1:** Amount of substance P and reactive oxygen species (ROS) in plasma following oil smoke exposure (OSE)

	Control (n = 8)	OSE (n = 8)	L733060+OSE (n = 8)	Lovastatin+OSE (n = 8)	Vitamin E+OSE (n = 8)	Catechins+OSE (n = 8)	P-value^
Substance-P (pg/mL)							
1 h after OSE	1.83 ± 0.44	54.71 ± 9.40^a^	23.00 ± 4.58^b^	48.29 ± 9.44^a^	27.57 ± 4.63	24.71 ± 4.83	< 0.001*
8 h after OSE	1.82 ± 0.50	72.93 ± 7.31^a^	38.99 ± 6.21^a, b^	57.13 ± 7.94^a^	51.38 ± 6.74^a, †^	45.50 ± 5.42^a, †^	< 0.001*
24 h after OSE	1.96 ± 0.48	61.44 ± 6.69^a^	35.86 ± 4.61^a^	58.25 ± 6.20^a^	52.63 ± 6.82^a, †^	46.50 ± 5.72^a, †^	< 0.001*
Plasma ROS (counts/10 s)							
1 h after OSE	103.86 ± 8.08	966.29 ± 107.17^a^	642.43 ± 96.50^a^	730.89 ± 115.32^a^	597.14 ± 77.70^a^	522.57 ± 87.07	< 0.001*
8 h after OSE	91.63 ± 6.78	2103.13 ± 204.06^a, †^	954.75 ± 68.99^a, b^	1502.25 ± 129.07^a, †^	1169.38 ± 145.79^a, b, †^	922.25 ± 112.51^a, b, †^	< 0.001*
24 h after OSE	108.43 ± 8.76	1806.16 ± 111.21^a, †^	1270.14 ± 127.84^a, †^	1325.29 ± 133.98^a, †^	1193.43 ± 192.99^a, †^	1185.71 ± 109.51^a, †^	< 0.001*

^ P-values based on ANOVA test. Pair-wise multiple comparisons between groups determined using Scheffe's test.

* Statistical significance, *P *< 0.05

^a ^Significantly different from control group at the respective time interval

^b^Significantly different from OSE group at the respective time interval

^†^Significantly different from treatment results at 1 h after OSE

### Effects of treatments before oil smoke exposure

We studied the effect of OSE on the levels of plasma SP by using enzyme-linked immunoassay and on the levels of plasma ROS by using ROS-induced chemiluminescence. Table 1 shows the SP and ROS levels in plasma following 120 min of OSE and for the 4 pretreatment groups. There were highly increased levels of SP and ROS after OSE alone. The SP and ROS levels were lower when 1 μg/kg L733060, 4 mg/kg lovastatin, 2.5 mg/kg vitamin E, or 2.5 mg/kg catechins pretreatment was provided before OSE.

Compared to the control, at each time point there were statistically significant differences from control (all *P *= < 0.001) in both SP and ROS levels after OSE. Compared to the SP levels after OSE, all the pretreatments we used reduced the effect of OSE on SP; however, only the effect of pretreatment with 1 μg/kg L733060 was statistically significant. After pretreatment with 1 μg/kg L733060, OSE induced 58% less SP at 1 h, and 47% less SP at 8 h. Likewise, compared to the ROS levels after OSE, all the pretreatments we used reduced the effect of OSE on ROS. However, only at the peak effect (the 8 h time point) did pretreatment with either 1 μg/kg L733060, 2.5 mg/kg vitamin E, or 2.5 mg/kg catechins statistically significantly reduce the effect of OSE on ROS. OSE induced 55% less ROS at 8 h when pretreated with 1 μg/kg L733060, 44% less ROS with 2.5 mg/kg vitamin E, and 56% less with 2.5 mg/kg catechins.

### ROS lung damage indicated by HEL levels

**Figure 2:**
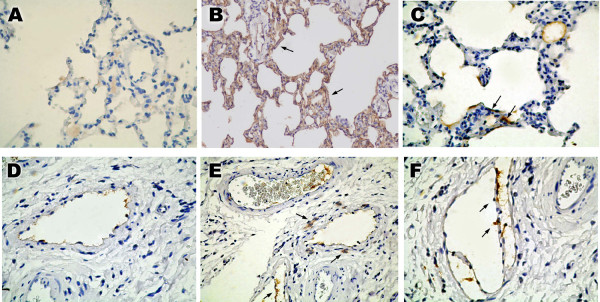
**Immunohistochemical evaluation of oil smoke exposure (OSE) on lung tissues**. Lung tissues were paraffin-embedded, sectioned, and stained brown with antibodies to VCAM-1 (A-C) or HEL (D-F). All images are × 200. (A) Endothelial cells in the capillaries of control rats (0 min OSE) did not stain for VCAM-1. (B) The endothelial cells of medium-sized vessels stained strongly for VCAM-1 after 120 min OSE. (C) When pretreated with the NK-1 receptor antagonist L733060, the endothelial cells in capillaries and medium- to large-sized vessels stained weakly for VCAM-1 after 120 min OSE. (D) Endothelial cells in the capillaries of control rats (0 min OSE) did not stain for HEL. (E) Endothelial cells in capillaries and lymphatic-like channels are positive for HEL after 120 min OSE. (F) When pretreated with L733060, endothelial cells in capillaries are positive and pneumocytes are negative for HEL after 120 min OSE.

Because we found that OSE increased ROS in BALF [7] and in plasma, we wanted to see if this was causing damage to the lung tissues. HEL is a marker for ROS damage; ROS oxidize lipids, and the resulting lipid peroxides modify proteins, creating HEL residues as a product. We immunohistochemically stained for HEL in lung tissues, and found that OSE produced HEL in and around the capillaries and lymphatic-like channels (Fig. 2D, E). After OSE, we also observed strong expression of VCAM-1, a molecule that recruits immune cells and is implicated in the generation of atherosclerosis (Fig. 2A, B). We have previously shown that OSE causes SP to interact with the NK-1 receptor and increase ROS [7]. Therefore we disrupted this process with 1 μg/kg of the NK-1 receptor inhibitor L733060, and we found that both less VCAM-1 and less HEL were induced after OSE (Fig. 2C, F).

**Figure 3:**
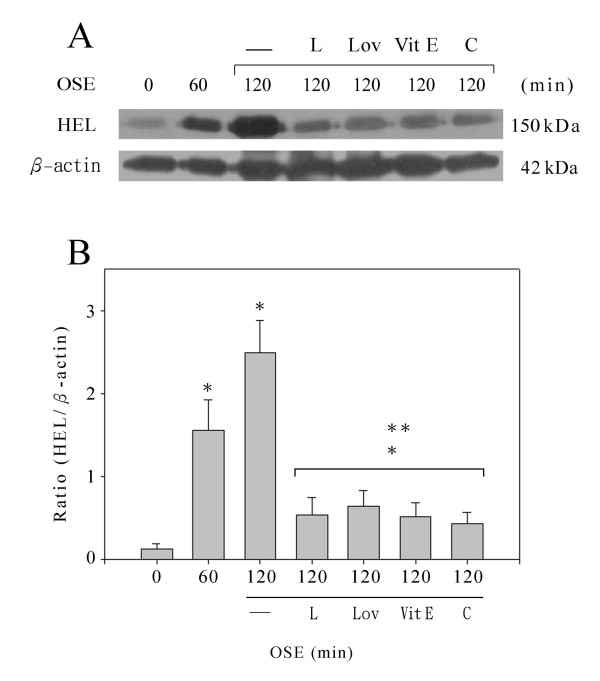
**Measurement of oxidative damage following oil smoke exposure (OSE)**. (A) Western blots were probed with a monoclonal antibody to HEL, an early and stable marker for lipid peroxidation derived protein oxidation. (B) The 150 kDa band from the HEL immunoblot was compared to the 42 kDa actin band by densitometry analysis. Mean and range of 8 replicate experiments are presented. The lanes contained protein samples from rat lungs exposed to 0, 60 or 120 min OSE, as well as samples from rats pretreated with L733060 (L), lovastatin (Lov), vitamin E (VitE), or catechins (C). * Indicates a significant difference from control rats with 0 min OSE. ** Indicates a significant difference from rats exposed to 120 min oil smoke without pretreatment; – Without drug pretreatment.

In addition to our qualitative microscopic observations, we investigated the effect of ROS after OSE by immunoblotting for HEL in western blots of total lung proteins. The western blots were probed with anti-β-actin as a baseline control and then with monoclonal anti-HEL antibody (Fig. 3A). In the western blot, the monoclonal antibody detected a 150 kDa band representing HEL-modified VLDL [21]. Using the 150 kDa HEL band and the 42 kDa actin band, the HEL/β-actin ratio was calculated from densitometry of the western blots as a quantitative measure of oxidative damage (Fig. 3B). In control rats that did not receive OSE, the HEL/β-actin ratio was 0.134. Exposing rats to oil smoke for 60 min increased the HEL to an HEL/β-actin ratio of 1.56, and to 2.49 at 120 min OSE. Pretreatment significantly decreased the HEL/β-actin ratio following 120 min OSE: 0.54, 1 μg/kg L733060; 0.65, 4 mg/kg lovastatin; 0.52, 2.5 mg/kg vitamin E; 0.43, 2.5 mg/kg catechins.

### Effect of OSE on thrombosis

Rats were given 120 min OSE and then used in the experimental thrombosis test at different times after the OSE treatment. We wanted to see if the increases in ROS that we observed after OSE correlated with a change in thrombosis. OSE induced a significant increase in the cumulative mass of thrombus generated in vivo at 0 (350%), 4 (354%), 8 (667%), 12 (583%) and 24 h (401%) compared with the thrombi induced in control rats at each time point (all *P *< 0.05). The peak of thrombus formation was at 8 and 12 h after OSE. These were significantly different than thrombus formation at 0 h after OSE (*P *< 0.05), corresponding with the statistically significant peaks of SP and ROS at 8 h following OSE (Table 1).

Thrombus formation was also studied in rats pretreated with inhibitors and antioxidants before 120 min OSE (Table 2). All pretreatments reduced the size of thrombus induced. Notably, 4 mg/kg lovastatin was least effective at reducing SP and ROS levels (Table 1) but was most effective at reducing thrombus formation (Table 2). At 1 h after OSE, the rats pretreated with 4 mg/kg lovastatin had no significant difference from control rats in the size of experimental thrombus formed. The pretreatments were all less effective at reducing thrombus formation at later time points. At 24 h after OSE thrombus formation in the pretreated rats was not significantly different than rats receiving OSE alone.

**Table 2:** Amount of thrombosis induced in rats 1, 8, or 24 h following oil smoke exposure (OSE)

	Control (n = 8)	OSE (n = 8)	L733060+OSE (n = 8)	Lovastatin+OSE (n = 8)	Vitamin E+OSE (n = 8)	Catechins+OSE (n = 8)	P-value^
Thrombus size (A.U.)							
1 h after OSE	10659 ± 695	45562 ± 3470^a^	29778 ± 3366^a, b^	22855 ± 2525^b^	31712 ± 3751^a^	26903 ± 2380 ^a, b^	< 0.001*
8 h after OSE	10509 ± 783	65794 ± 4327^a, †^	38742 ± 4999^a, b^	27660 ± 2052^a, b^	35589 ± 2579^a, b^	36541 ± 3359^a, b^	< 0.001*
24 h after OSE	10458 ± 709	52416 ± 3191^a^	41574 ± 4772^a^	36955 ± 4246^a, †^	49068 ± 4881^a, †^	38707 ± 5560^a^	< 0.001*

^ P-values based on ANOVA test. Pair-wise multiple comparisons between groups determined using Scheffe's test.

* Statistical significance, *P *< 0.05

^a ^Significantly different from control group at the respective time interval

^b^Significantly different from OSE group at the respective time interval

^†^Significantly different from treatment results at 1 h after OSE

Table 3 shows the amount of platelets, RBCs, WBCs and fibrinogen after 120 min OSE. After 120 min OSE, rats receiving OSE alone showed significantly different increases from control rats in platelets, RBCs, WBCs and fibrinogen. All pretreatments reduced the platelet, RBC, WBC and fibrinogen levels (Table 3). Pretreatment with 4 mg/kg lovastatin was highly effective at increasing WBC count from control rats. Moreover, WBC in the rats pretreated with 2.5 mg/kg catechins were significantly different than WBC in rats receiving OSE alone And, fibrinogen in rats pretreated with 4 mg/kg lovastatin was significantly different than WBC in rats receiving OSE alone.

**Table 3:** Amount of platelet, RBC, WBC and fibrinogen after 120 min oil smoke exposure (OSE)

	Control (n = 8)	OSE (n = 8)	L733060+OSE (n = 8)	Lovastatin+OSE (n = 8)	Vitamin E+OSE (n = 8)	Catechins+OSE (n = 8)	P-value^
Platelet (×10^3^/μL)	103.88 ± 18.30	410.63 ± 45.30^a^	223.13 ± 41.11	188.25 ± 20.51	268.00 ± 73.58	257.00 ± 47.70	0.001*
RBC (×10^6^/μL)	1.54 ± 0.31	5.03 ± 0.75^a^	3.24 ± 0.47	3.11 ± 0.49	2.91 ± 0.50	2.68 ± 0.35	0.001*
WBC (×10^3^/μL)	1056 ± 105	5722 ± 693^a^	3403 ± 465	4127 ± 617^a^	3240 ± 605	3088 ± 409^b^	< 0.001*
Fibrinogen (mg/dl)	48.88 ± 9.00	340.00 ± 49.89^a^	213.00 ± 38.08	157.88 ± 26.90^b^	176.75 ± 34.33	224.13 ± 45.09	< 0.001*

^ P-values based on ANOVA test. Pair-wise multiple comparisons between groups determined using Scheffe's test.

* Statistical significance, *P *< 0.05

^a ^Significantly different from control group

^b^Significantly different from OSE group

## Discussion

This study explored the effects of APM on rats by exposing them for a short period of time to lard oil smoke, a type of APM exposure that might be normally encountered by people, especially when cooking. By pretreating the rats with single doses of various inhibitors and antioxidants, we explored the mechanisms involved in the physiological response to oil smoke exposure. Because we only used single doses of each pretreatment (rather than generating dose-response curves), no conclusions can be drawn about the relative efficacy of each type of pretreatment. This study is limited to general conclusions about the mechanisms underlying the physiological responses to oil smoke APM.

Smoking (both active and passive) increases the cardiovascular risk of atherosclerosis and thrombosis. Vascular inflammation and oxidative stress caused by inhalation of cigarette smoke are thought to be initiating events that lead to these two outcomes. The reactive oxygen species generated during oxidative stress oxidize low density lipoprotein, a reaction that makes LDL more easily taken up by macrophages, which are then deposited as foam cells in atherosclerotic plaques in the arteries [22]. If the plaques rupture, the tissue factor on the macrophages in the plaques comes in contact with clotting factors in the blood and thrombosis results. There is also a body of evidence suggesting that smoking up-regulates the coagulation system, thus making it easier for any pro-coagulatory stimulus to cause thrombus formation.

Many lines of evidence attribute the initiation of atherosclerosis to a complex series of events and circulating factors acting on immune cells and the smooth muscle and endothelial cells making up the blood vessels. Here and in our previous paper we demonstrated that insult to the lungs by oil smoke was followed by an increase in SP levels in bronchoalveolar lavage fluid (BALF) [7] and plasma. The SP may have been released from sensory nerve endings in response to the oil smoke particles [23]. After OSE, ROS levels also rose along with the rise in SP levels in BALF [7] and in plasma. OSE causes interstitial pneumonitis in rat lung tissue, thickening of the alveolar-capillary membrane, and a large increase in inflammatory cells and dying epithelial cells [7]. Inhibiting SP, inhibiting the NK-1 receptor, or using antioxidants reduced the effects of OSE. This suggests that OSE stimulates SP release which, acting via the NK-1 receptor, attracts leukocytes which generate ROS.

Signs of leukocyte recruitment after OSE include histological observation of increased neutrophils, monocytes and leukocytes in lung tissue [7], as well as increased expression of the endothelial surface proteins intercellular adhesion molecule-1(ICAM-1) [7] and VCAM-1, which recruit inflammatory cells. Inflammatory recruitment is a step in the progression of early atherosclerosis. Increases in molecules such as ICAM-1 and VCAM-1 may also be important with regard to smooth muscle cells [24]. Both the smooth muscle cells and endothelial cells lining vessels can absorb damaged lipids and become foam cells [25]. The ICAM-1 and VCAM-1 molecules stabilize these cells against apoptosis which helps form the initial accumulation of cells of an atherosclerotic plaque [24]. Likewise, lovastatin can reduce oxidative damage to mouse lungs during reperfusion injury and reverse upregulation of VCAM-1 [26], and catechins can reduce SP-mediated ROS and ICAM-1 expression in irritated bladders [13].

Atherosclerotic plaques stain strongly for HEL [27], and we found that OSE stimulated the formation of HEL in lung tissues, an effect that was reversible by inhibiting the SP pathway with the NK-1 receptor antagonist, lovastatin or antioxidants. Other studies have come to similar conclusions about the connection between smoke particle inhalation and lung tissue damage. Yasuda et al. exposed mice to diesel exhaust particles (DEP) and argued that the particles generated ROS; they observed a number of DNA, protein and lipid damage products (including HEL) that are attributable to ROS because they could be prevented by antioxidants [28]. They also observed an increase in VCAM-1 and ICAM-1. Their results agree with ours, but we demonstrate further that the mechanism involved is not the direct generation of ROS by combustion particles, but the generation of ROS following SP/NK-1R signaling and an inflammatory response. Arguing against the importance of the type of particles is an experiment in which hamsters inhaled 400-nm polystyrene particles; these hamsters also developed inflammation, released histamine and had enhanced thrombosis [17].

In hamsters, diesel exhaust particles cause platelet activation and enhance experimental thrombosis [18]. The DEP exposure increases platelet activity, and increases the histamine and the number of neutrophils in the hamster BALF; inhibiting the histamine H1 receptor reduces the neutrophilia but does not affect the prothrombotic effects [18]. In conjunction with our results from oil smoke exposure in rats, we can propose that the DEP exposure in hamsters caused release of SP, which was directly involved in the prothrombotic effect. The increase in neutrophils, and possibly other inflammatory cells, may involve a pathway instigated by SP that is dependent upon histamine.

Acute exposure to APM, such as exposure to cooking oil smoke, can lead to increased numbers of activated white bloods cells, which can lead to increased NADPH oxidase activity and add to the risk of coagulation/thrombosis, development of atherosclerosis, and atherosclerotic plaque vulnerability [4]. We found increased white blood cells following acute oil smoke exposure, as well as increased platelets and fibrinogen (Table 3). The increased platelets and fibrinogen could be responsible for increased thrombosis [4]. Jones et al. [15] show that platelets express NK-1R and that substance P promotes thrombosis. Inhibition of NK-1R reversed this effect [15]. These results provide a mechanism to explain why thrombosis was affected by OSE in our experiments. Jones et al. suggest that NK-1R antagonists may be useful antithrombotic drugs.

Because substance P has many functions, it remains a question what the beneficial function of SP is in response to combustion particles. It is possible that substance P is only incidentally promoting platelet activation and ROS generation in addition to some beneficial effect. Further exploration of the detailed mechanisms following smoke particle exposure are needed to determine which inhibitors (such as SP inhibitors, NK-1R inhibitors, or antioxidants) are appropriate to use to combat the unwanted side effects without preventing potential protective functions of SP stimulation. Smoke inhalation is connected with carcinogenesis, and the SP-induced attraction of immune cells may help collect and destroy the particulate matter. But, the attracted immune cells generate ROS, which are damaging, and the increased SP may also activate platelets and promote foam cell accumulation, predisposing to atherosclerosis and myocardial infarction.

The temptation to use NK-1R inhibitors, such as L733060, as antithrombotics [14] should be approached with caution because they can also cross the blood-brain-barrier and affect the neurological functions of SP [29-31]. It might be worthwhile to investigate alternate targets in the pathway, such as DPPIV, which interacts with the SP/NK-1R inflammatory pathway in allergic rhinitis [32].

## Conclusion

Exposure to APM is associated with increased risk of atherosclerosis and myocardial infarction. This study adds to the literature on the mechanism connecting APM to these health risks. OSE stimulated an increase in SP in the plasma, which in turn seems to have recruited inflammatory cells that generate ROS, as evidenced by increases in endothelial attachment molecules (VCAM-1). We measured increases in HEL, showing that the ROS damaged lipids and proteins after OSE. HEL and other oxidative lipid damage can promote lipid accumulation by macrophages and smooth muscle cells to form foam cells, which collect into early atherosclerotic plaques. Thus, oil smoke exposure had two main outcomes: 1) ROS Damage (due to SP recruiting inflammatory cells, and the inflammatory cells generating ROS, leading to immediate lipid damage, and then foam cells, and then long-term accumulation of atherosclerotic plaque); and 2) thrombosis (due to SP activating platelets via the NK-1 receptor). These two separate pathways could converge again when thrombosis causes the rupture of existing atherosclerotic plaques and causes myocardial infarction.

## Competing interests

The authors declare that they have no competing interests.

## Authors' contributions

Chang Li-Ching and Chen Wen-Chung carried out the immunoassays. Lin Yu-Ching participated in the sequence alignment. Li Ping-Chia participated in the design of the study and performed the statistical analysis. Lin Yu-Ching conceived of the study, Li Ping-Chia and Chang Li-Ching participated in its design and coordination and helped to draft the manuscript. All authors read and approved the final manuscript.
